# Loneliness and trust issues reshape mental stress of expatriates during early COVID-19: a structural equation modelling approach

**DOI:** 10.1186/s40359-023-01180-9

**Published:** 2023-04-29

**Authors:** Md Arif Billah, Sharmin Akhtar, Md. Nuruzzaman Khan

**Affiliations:** 1 Faculty of Business, Economics and Social Development, Universiti Malaysia Terengganu, 21030 Kuala Nerus, Terengganu, Malaysia; 2 Health System and Population Studies Division, icddr,b, Dhaka, 1212 Bangladesh; 3 Department of Population Science, Jatiya Kabi Kazi Nazrul Islam University, Trishal, Mymensingh, Bangladesh

**Keywords:** Perceived stress, Perceived loneliness, Interpersonal trust, Institutional trust, Structural equation modelling, Expatriates, Global

## Abstract

**Objectives:**

To explore relationship among perceived stress regarding loneliness, interpersonal trust and institutional trust of expatriates during the early COVID-19 period (from 30^th^ March to 30^th^ May 2020).

**Methods:**

Data from  21,439 expatriates were extracted from COVIDiSTRESS global survey. The outcome variable was perceived stress. The explanatory variables were age, perceived loneliness, trust (interpersonal and institutional). Pairwise correlation, and structural equation modelling were used to determine relationship among outcome and explanatory variables.

**Results:**

The majority of the expatriates were female (73.85%), married (60.20%), had college degree (47.76%), and employed (48.72%). Over 63% of the total expatriates reported that the COVID-19 pandemic changed their lives. The average age of the respondents was 40.4 years (± 13.7), and the average score of perceived stress, loneliness, interpersonal and institutional trust were 25.5, 7.4, 14.2 and 40.4, respectively. We found a moderate correlation of perceived stress with age, perceived loneliness, interpersonal trust and institutional trust (*p* < 0.001). They were also found moderately related to each other. Structural equation modelling evaluated that a lack of trust can cause loneliness among expatriates, which later lead to perceived stress. Interpersonal trust was more likely to be associated with stress than institutional trust, whereas perceived loneliness mediated between both trusts and perceived stress.

**Conclusion:**

Perceived stress can be reduced through trusting others and alleviating the loneliness. Making strong linkage among migrants as well as between migrants and local community is important to ensure proper mental wellbeing of expatriates.

**Supplementary Information:**

The online version contains supplementary material available at 10.1186/s40359-023-01180-9.

## Introduction

The twenty-first century’s globalization has resulted in an increase in cross-border assignments, facilitating international collaboration among individuals worldwide. However, due to the worldwide spread of COVID-19 pandemic, countries enforced border closures and lockdowns, with people being instructed to stay at home. Public places such as schools and colleges were shut down, and employees were required to work remotely. Consequently, social and interpersonal distance were created, leading to increased loneliness and reduced trusts. These factors have had a detrimental impact on the mental well-being of individuals in various socioeconomic situations, with expatriates being particularly affected. [1, 2].

## Background of the study

The term "perceived loneliness" relates to a lack of social relationships and reduced social interactions [3]. Loneliness is also defined as the discrepancy between individual’s desired and actual relationships with others and the environment, which is resulted in lack of trust and self-isolation [4–7]. However, loneliness can also result from self- or social alienation due to isolation, emotional pain, and cognitive development [26]. This is a subjective feeling that can cause unpleasant and distressing situations [7]. Furthermore, inadequate social connections, including connection with the close personnel and institutions whether they were engaged with or not, can exacerbate feelings of loneliness [8, 9]. This form of loneliness is situational and transient, varying over different chronic periods, such as during a pandemic [9–12]. Lonely individuals are more sensitive to social threats due to the consequences of their loneliness, which causes a lack of trust and paranoia [12–15]. The evolutionary theory of loneliness suggests that it is an adaptive mechanism that produces an “aversive state” eliciting social pain, unsafe feelings, and biased negative social information [12, 16]. Moreover, individual’s loneliness can disrupt their social bonds and activate mental chaos in their mind [16, 17]. Therefore, individual with perceived loneliness often feel low level of self-esteem and optimism. Later that resulted in several distressful events and causes perceived stress [9, 12]. These behaviours can lead to mental disorders and suicidal tendencies [18]. A recent review found that loneliness resulting from social isolation can increase the mortality by 29–32% [19].

Trust plays vital role in social interactions and is critical aspect of personal relationship [20, 21]. Perceived loneliness also incorporates trust issues in the form of social connectedness as it connotes sensitivity to social contexts [5]. Insufficient social connectedness is raised from the lack of faith in close persons/interpersonal trusts and institutions where they worked (institutional trust), boosts the experience of loneliness [8, 9]. Researchers have identified two types of loneliness: emotional loneliness, which involves an absence of connection and trust in others; and social loneliness, which is characterized by perception of a lack of interpersonal connections or a deficient social network [8]. Studies lead by Rotenberg support these loneliness classifications by adopting the mechanism of cognitive schema and social disengagement which incorporates the trusts of individuals. Their studies suggested that the negative form of trusts can affect the socio-emotional behaviour and elevated the state of loneliness (cognitive schema) or incorporate to the ineffective social interactions and reduced the social relationships (social disengagement) [4, 22–24]. Therefore, individuals' trust in forms of social disengagement and cognitive attachment can thus be negatively correlated with loneliness.

Expatriates (or expats) are experiencing higher cultural diversity than others because of community-level objective factors and cultural parameters, including communication trusts [25–27]. They optimized the cross-cultures to serve their practical needs, from survival to maintaining order, socio-economic viability, and communication patterns [28, 29]. As a result, they primarily address socio-cultural and psychological adjustments for mental well-being. It includes negotiation with the organizational and environmental trust or daily stress in the host culture [30, 31]. There is evidence that expatriates felt lonely when they faced acculturation and adaptation problems in the host country [32]. Besides, COVID-19 enhanced individuals' levels of isolation, integrated with socio-cultural isolation, and impacted their psychosocial wellbeing, perceived loneliness, and stress [33, 34]. One study found that social disconnectedness and increased stressors during the pandemic reduced trust in government institutions [33].

**Fig. 1:**
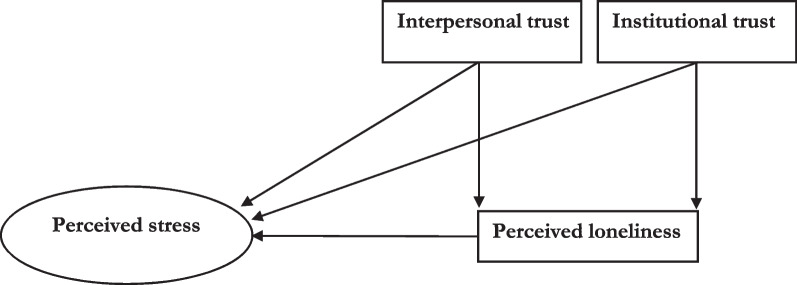
Conceptual framework and hypothesis

All these models indicate that the trust issues of a person can contribute to their stress and loneliness. This emphasizes a socio-cognitive perspective on loneliness and suggests that cognitive stresses of a person affected by loneliness and the lack of trust manifested in their behaviours [4, 9, 12, 13, 16]. The previous literature on expatriates in intercultural psychology loosely examined the experiences of loneliness, stress, and trust issues across culture [27]. However, this relationship has yet to be clearly explained in long-term, non-aversive situations, which is particularly relevant during the COVID-19 pandemic. To address this gap, we developed a conceptual model of perceived stress, loneliness, and trust, drawing on previous literature and incorporating theories of loneliness and dynamics of attachment theory (See Fig. 1). Although this relationship has not been fully elucidated for expatriates, our study focuses on evaluating perceived stress related to loneliness, interpersonal and institutional trust for individuals crossing cultures and borders, especially during the COVID-19 pandemic.

## Methods and materials

### Study overview

We utilized data from the collaborative COVIDiSTRESS global survey (COVIDiSTRESS global survey network, 2020—available at https://osf.io/2ftma/) which aimed to improve our understanding of human experiences during the COVID-19 pandemic from a psycho-social perspective. The primary respondents of this survey were people who were living outside of their country of origin or a in a foreign country (i.e., their country of residence is not their birthplace). The survey was conducted from March 30 to May 30, 2020 through an open science forum, which was an online platform. A total of 173,426 respondents voluntarily participated from 179 countries. The survey was conducted in 47 different languages and dialects with collaborators from 39 countries and regions. The dataset includes background demographic information as well as measures of the perceived stress (PSS-10), perceived loneliness (PLS-3), and trusts, and so on. Details of the sampling procedure have been published elsewhere [35].

### Sample

**Fig. 2:**
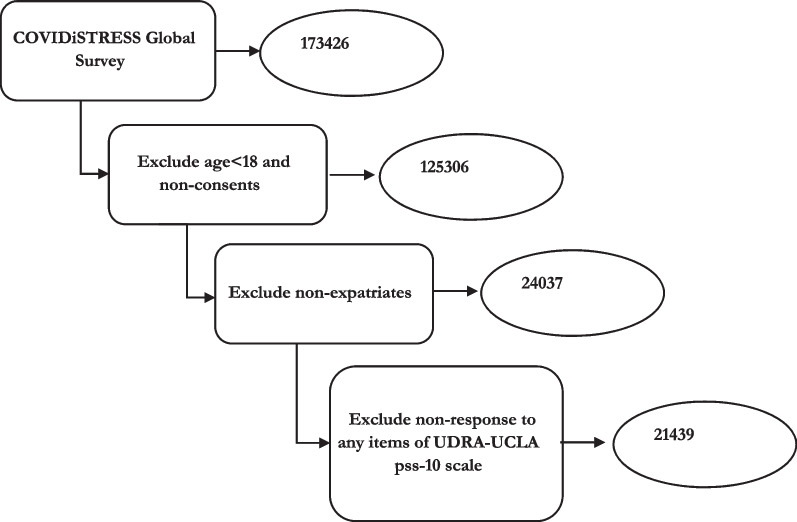
Sample selection process

This study included 21,439 respondents (See Fig. 2) following three inclusion criteria- (i) age 18 years or older, (ii) lived outside their country of origin (Who responded yes to the question “Are you currently living outside of what you consider your home country?”), and (iii) were completely respond to perceived stress items (PSS-10).

### Ethical approval

The data for COVIDiSTRESS was collected online using the Qualtrics platform from individuals who participated voluntarily and gave informed consent. The survey was pre-registered as a project on the OSF platform (details given at https://osf.io/6ut4v). The data collection was anonymous, and ethical clearance was waived by the IRB legal department at Aarhus University, Denmark to allow for urgent data collection. No additional ethical approval is required for this study.

### Variables and measures

Perceived stress (PSS-10): The perceived stress of individual was defined by the perceived stress scale (PSS-10) comprises 10 items with a 5-point Likert scale (see Additional file 1) ranging from 1 (Never) to 5 (Very often). Among the items, four items (items 4,5,7, and 8) were scored in reverse, as they were positively stated [36]. The perceived stress score was calculated by summing all items, which range from 10 to 50. Higher scores indicate greater perceived stress. The Cronbach alpha of 0.8736 indicated good and acceptable reliability.

Perceived loneliness (PLS-3): The perceived loneliness of the respondents was defined by the three items of perceived loneliness scale (PLS-3) with a 5-point Likert scale (see Additional file 1) ranging from 1 (Never) to 5 (Very often). The score for an individual’s perceived loneliness was obtained by summing these items [37]. Higher scores indicate a higher level of perceived loneliness. The Cronbach alpha of 0.7448 indicated the accepted range of reliability.

Trust issues: Trust issues were divided into two categories: interpersonal trust and institutional trust. Each item was recorded on a scale of 10 equivalent distances (see Additional file 1) ranging from 0 (do not trust) to 10 (have complete trust). Interpersonal trust has two items, which were combined to create a composite score ranging from 0 to 20. Institutional trust has six items, which were combined to create a composite score ranging from 0 to 60. In both cases, the Cronbach alpha showed acceptable reliability of interpersonal trust (0.7612) and institutional trust (0.8959).

### Statistical analysis

Frequency distribution was reported for the sample characteristics of group data. Descriptive statistics, including the mean, standard deviation and ranges, were used for the continuous variables. Pairwise correlation coefficients were estimated for those continuous variables at < 5% and < 1% level of significance. In pairwise correlation analyses, continuous variables-age, perceived stress, loneliness, and trusts were included to see the correlations between each other. This also indicates the multicollinearity which is moderate. Besides, variance inflation factor (VIF) was used to see the multicollinearity.

Structural equation modelling (SEM) was used to confirm the relationships among the perceived stress, loneliness and interpersonal and institutional trust. The maximum likelihood estimation method (mlmv) was used in the SEM analysis, taking into account measurement errors. In the model, the PLS attribute was enacted as the mediator and exogenous variable, while the institutional and interpersonal trust were the exogeneous variable for perceived stress (endogenous variable) [38]. As chi-square is largely affected by the sample size, we have used several goodness fit indices of SEM for model evaluation, including the likelihood ratio (Chi-square/df), the comparative fit index (CFI), Tucker-Lewis fit index (TLI), the goodness of fit index (GFI), and root mean square error of approximation (RMSEA) [39]. The acceptable threshold for RMSEA was ≤ 0.06, that of χ^2^/df was < 3, and that of the rest of the indices, such as CFI, was ≥ 0.95 [40]. Furthermore, we also examine the statistical significance of the fitted model’s direct, indirect and total effects. All analyses were conducted in statistical software STATA 14 (StataCorp, 2015).

## Results

### Sample characteristics

Reported average age of the expatriates was 40.39 years (SD, 13.72) (Table 1). A majority of the expatriates were female (73.85%) and had college-level degrees (77.77%). Around half of the total expatriates reported they engaged with a full-time employment (48.72%). Nearly two-third of the total expatriates were married/cohabiting (60.20%) at the time of the survey. Around 63% of the total expatriates reported they were at risk of getting the COVID-19, though around 58% were put themselves in minor isolation and  37% put themselves in complete isolation.

**Table 1:** Sample characteristics of the expatriates

Variables	n (%)	$\overline{x }$(*SD)*, Range
Age		40.39 (13.72), 18–110
Gender		
Male	5407 (25.22%)	
Female	15,833 (73.85%)	
Prefer not to say/others	199 (0.93%)	
Education		
Up to 12-year schooling	2446 (11.41%)	
College, bachelor and master	10,240 (47.76%)	
College/short cont. education	6434 (30.01%)	
PhD./Doctorate	2108 (9.83%)	
Other (Uninformative/NA/None)	211 (0.98%)	
Employment		
Not employed	1775 (8.28%)	
Part-time employed	2171 (10.13%)	
Self-employed	2114 (9.86%)	
Full-time employed	10,445 (48.72%)	
Student	3074 (14.34%)	
Retired	1701 (7.93%)	
NA	159 (0.74%)	
Marital status		
Single	6237 (29.09%)	
Married/cohabiting	12,907 (60.20%)	
Divorced/widowed	1524 (7.11%)	
Other (uninformative/NA)	771 (3.59%)	
COVID-19 risk		
Yes	13,602 (63.45%)	
No	6092 (28.42%)	
Not sure/NA	1745 (8.14%)	
Isolation type		
No isolated	767 (3.58%)	
Minor isolated	12,485 (58.23%)	
Complete isolated	8000 (37.32%)	
Others (uninformative/NA)	187 (0.87%)	
Perceived stress		25.48 (7.25), 10–50
Perceived loneliness		7.38 (2.86), 3–15
Interpersonal trust		14.15 (3.36), 0–20
Institutional trust		40.40 (12.37), 0–60
Overall trust		54.64 (14.28), 0–80

NA- Not available, n- frequency, %- percentage, $\overline{x }$- mean, SD- standard deviation.

The mean score of perceived stress, perceived loneliness, interpersonal trust and institutional trust were 25.48 (SD, 7.25), 7.38 (SD, 2.86), 14.15 (SD, 3.36) and 40.40 (SD, 12.37), respectively. The mean overall trust score was 54.64 (SD, 14.28).

### Correlation matrix

The expatriate’s perceived stress (PSS) was found to be negatively correlated with age (r = − 0.29, *p* < 0.001), interpersonal trust/trust in people (IPT) (r = − 0.31, *p* < 0.001), institutional trust/trust in institution (IT) (r = − 0.25, *p* < 0.001) while positively correlated with their perceived loneliness (PLS) (r = 0.57, *p* < 0.001) (See Table 2). PLS found negatively correlated with age (r = − 0.20, *p* < 0.001), IPT (r = − 0.21, *p* < 0.001) and IT (r = − 0.16, *p* < 0.001). Expatriate’s age was found positively correlated with IPT (r = 0.23, *p* < 0.001) and IT (r = 0.12, *p* < 0.001). The IPT and IT was found positively correlated to each other (r = 0.49, *p* < 0.001).

**Table 2:** Pairwise correlation estimates

Variables	(1)	(2)	(3)	(4)	(5)
(1) Age (r)	1.00				
(N)	21,439				
(2) Perceived stress (r)	− 0.29***	1.00			
(N)	21,439				
(3) Perceived loneliness (r)	− 0.20***	0.57***	1.00		
(N)	21,301	21,301			
(4) Interpersonal trust (r)	0.23***	− 0.31***	− 0.21***	1.00	
(N)	20,613	20,613	20,483		
(5) Institutional trust (r)	0.12***	− 0.25***	− 0.16***	0.49***	1.00
(N)	19,898	19,898	19,774	19,811	

***indicates the *p* < 0.001, r-correlation coefficients, N-number of observations

### Structural equation modelling

**Fig. 3:**
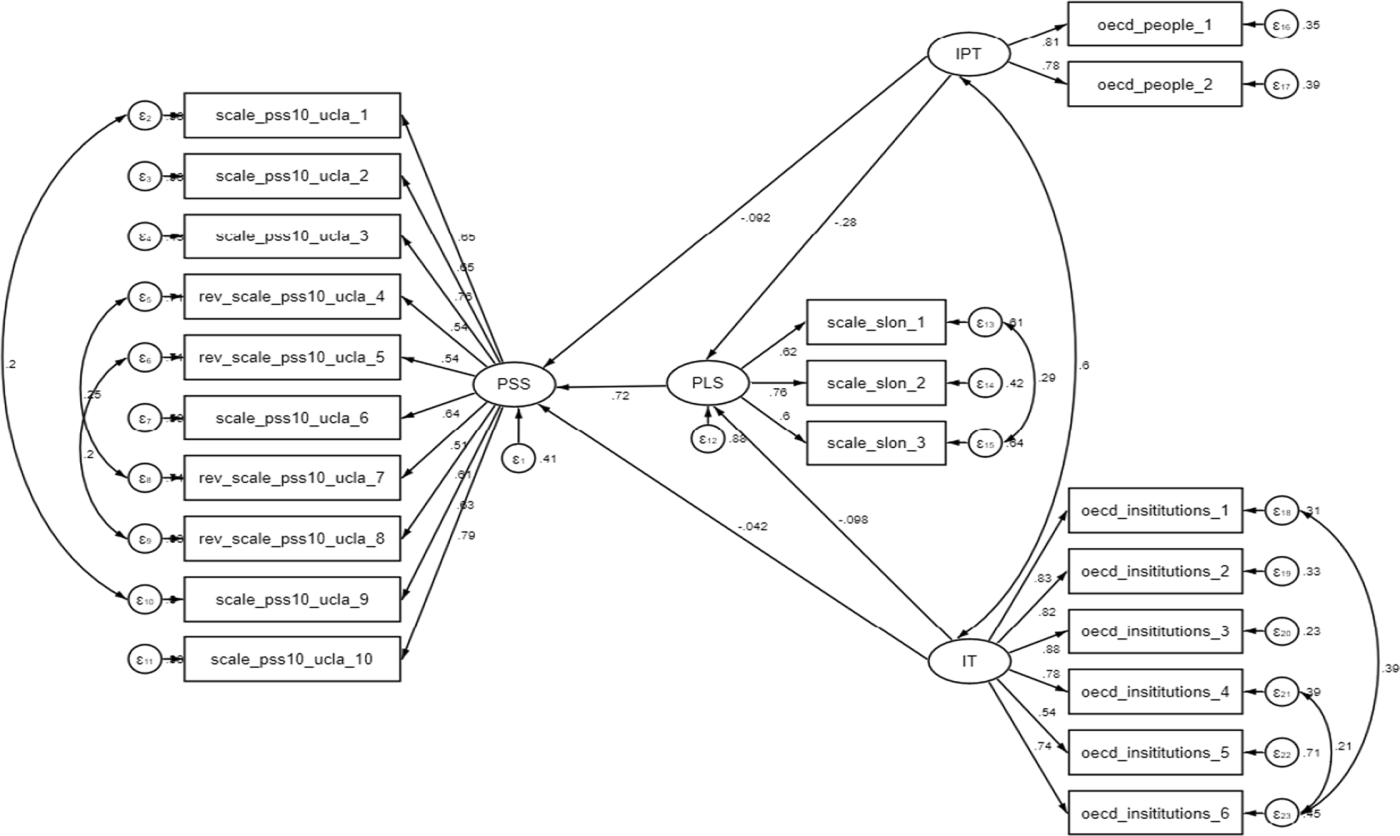
Structural equation model indicating the path how the interpersonal trust (IPT), institutional trust (IT) and perceived loneliness (PLS) related to the perceived stress (PSS) of people who were living abroad during the early pandemic (standardized estimates were reported)

We found a significant effect on perceived stress from loneliness (ρ < 0.001), interpersonal trust (ρ < 0.001) and the interaction between loneliness and interpersonal trust (ρ < 0.05) (See Fig. 3). Additionally, perceived loneliness had a direct and positive contribution to perceived stress (Std Coeff, 0.72; SE, 0.012), while interpersonal trust (Std Coeff, − 0.28; SE, 0.006) and institutional trust (Std Coeff, − 0.10; SE, 0.004) had a direct negative contribution to perceived loneliness (see Table 4). Interpersonal trust (Std Coeff: − 0.09; SE, 0.004) and institutional trust (Std Coeff, − 0.04; SE, 0.003) also had negative impacts on perceived stress among expatriates during the early stage of the COVID-19 pandemic. We also found an indirect but negative effect of interpersonal (Std Coeff, − 0.20; SE, 0.004) and institutional trust (Std Coeff, − 0.07; SE, 0.003) on perceived stress. The total impact effect of interpersonal (Std Coeff, − 0.29; SE, 0.004) and institutional trust (Std Coeff, − 0.11; SE, 0.003) also negatively impacted perceived stress. Overall, the model fitting statistics (Table 3) indicated a good fit of the model based on the *RMSEA* (0.04), *CFI* (0.96), *TLI* (0.96) and *GFI* (0.96).

**Table 3:** Overall fitted results of structural equation modelling using the maximum likelihood method with missing values^1,2^ (n = 21,439 expatriates)

Fit statistics	Estimates		Reference^5^
Likelihood ratio			
Chi-square MS (177)	7517.49		
p > chi2	0.000		
Chi-square/df	47.18		< 3.00
Chi-square BS (210)	197,829.15		
Population error			
RMSEA	0.04		< ≈0.06
90% CI	0.043–0.045		
Pclose	1.000	P(RMSEA) < = 0.05	
Information criterion			
AIC^3^	1,391,196.27		
BIC^3^	1,391,794.24		
Baseline comparison			
CFI	0.96		> 0.95
TLI	0.96		> 0.95
GFI	0.96		
Size of residuals			
CD	0.972		
SRMR^4^	Not reported		

MS- model vs saturated; BS- baseline vs saturated; RMSEA- root mean squared error of approximation; CI- confidence interval; CFI- Comparative fit index; TLI- Tucker-Lewis index; GFI- goodness of fit index; CD- coefficient of determination and SRMR- standardized root-mean-square residuals

^1^–Method: mlmv; Log-likelihood: –695,523.13

^2^Endogenous latent variable: PSS and exogenous latent variables: PLS, IPT and IT

^3^Akaike's and Bayesian information criteria (AIC and BIC) were ignored (Huang, 2017)

^4^SRMR is not reported due to missing values

^5^Reference values were provided in the third column (Hu & Bentler, 1999; Kline, 2015)

**Table 4:** Direct, indirect and total effects

	Direct effect	Indirect effect	Total effect
	Std coef (SE)	Std coef (SE)	Std coef (SE)
PSS ← PLS	0.72 (0.011)***		0.72 (0.012)***
PSS ← IPT	− 0.09 (0.004)***	− 0.20 (0.004)***	− 0.29 (0.005)***
PSS ← IT	− 0.04 (0.003)***	− 0.07 (0.003)***	− 0.11 (0.003)***
PLS ← IPT	− 0.28 (0.006)***		− 0.28 (0.006)***
PLS ← IT	− 0.10 (0.004)***		− 0.10 (0.004)***

*** indicates 0.01% level of significance, Std coef: standardized coefficients, SE: standard error, PSS: perceived stress, PLS: perceived loneliness, IPT: interpersonal trust, and IT: institutional trust.

## Discussion

The present study investigated the role of perceived loneliness and trust issues, both interpersonal and institutional, on perceived stress among expatriates during the early COVID-19 pandemic. The study found a relationship among perceived stress, loneliness, and interpersonal and institutional trust. Our fitted model supports the conceptual framework that expatriate’s loneliness and trusts directly affect their mental stress, while their lack of trusts in other persons and institutions directly cause mental stress and indirectly impacts stress through loneliness. Therefore, the lack of trusts of an expatriate cause perceived loneliness, which later increase their perceived stress.

We found that expatriate’s low levels of trusts, especially in interpersonal relationships, can lead to increased loneliness. Similar findings have been reported in studies on university students [41] and married couples [42]. This relationship is typically based on the degree of attachment to the person or institution of interest. Insecure attachment can transform self-centred preferences into pro-relationship motivations, and this type of motivation is mainly inferred from interpersonal trust [20]. A multi-modal study had demonstrated that individuals with acute level of loneliness have significantly less interpersonal trust compared to non-acute loners [43]. They are less likely to engage in social interactions because their safety behaviours increase interpersonal distances [13]. This phenomenon is mostly prevalent among marginalized individuals such as expatriates, who have a weakened social immune system and social inflammation [9, 44, 45]. All of these social stimuli sensitize expatriates to avoid hostile individuals and align themselves with trusted ones when vulnerable [45]. Therefore, integrated and compromised trusts effectively contribute to expatriate’s loneliness and ineffective social interactions [43] and social disengagement[5, 46]. For instance, in the case of older people, when neighbours are trustworthy, loneliness reduces significantly [47]. Interpersonal trust interacts with and mediates social and family loneliness[48]. Interpersonal trust mediated between attachment and relationship quality, whereas relationship quality mediated between loneliness and attachment [42]. In this circumstances, wide access to the technology and online connectedness with the close one can be buffer this low level of trust and feeling loneliness inside.

Our analysis found a reverse relationship between interpersonal trust and perceived stress due to perceived threat [49], negative social interactions [50] and psychological discomfort [51]. Studies have also shown that trust can moderate distressful events by eliminating those threats and discomforts [49, 52]. Higher levels of trust increase positive expectations and confidence in others, mitigating psychological discomfort [52]. Additionally, decreased community engagement is associated with depressive symptoms, while interpersonal trust is associated with increased social engagement [53]. Nickerson et al. (2019) found this relationship among settled refugees in other countries [54]. Although online engagement is promoted as a way to reduce loneliness by increasing trusted interactions, studies have shown that it is not a permanent solution [55, 56].

In our sample, most of the expatriates were students and labour who were dependent on their respective organizations. International organizations also collaborate with national agencies from various countries, channelling the most recent and up-to-date news while taking measures to reduce transmission and negative impacts. Expatriates mostly rely on these social, national, and international organizations, as well as their working places, exhibiting the organizational dynamics of attachments, including institutional trusts [57]. Furthermore, during the pandemic's lockdown and isolation, the relationship between students and their respective academic institutions was severely strained due to changing institutional regulations and restrictions over time, which created distinctive situations that made them feel disconnected from their academic institutions [58]. Institutional trusts can influence the stress and stress factors of expats by controlling individual and cultural diversifications. On the other hand, loneliness in the workplace resulted from a lack of trust in organizational leaders, as trust in leaders creates a space for social interactions within organizations [59].

The COVID-19 period causes a job recession, prioritizes locations over migrants, changed strategies and policies, defeated COVID-19, and restrained the organizational and political economy, which consistently influenced their trust in organizations, influencing their psychological distress [57]. We found institutional trusts to be more likely to influence an individual’s stress than loneliness. Slight relationships with loneliness occurred as a result of isolation, movement controls, and frequent decision changes, which caused expatriates to spend more time idle and alone [59, 60].

We found a positive, direct, and extended relationship between loneliness and stress. These variables mediate other biopsychosocial outcomes. Many recent studies have supported this relationship in the context of pandemics [55, 61–63]. During this pandemic, Landmann and Rohmann integrated the physical dimension of loneliness, which has become more prevalent due to preventive protocols. In their study, emotional and social loneliness predicted a low level of psychological wellbeing and enhanced stress factors, while physical loneliness significantly reduced positivity regarding mind and body, joyfulness, connectedness, and enhanced worries. Emotional loneliness resulted in physical distance and a loss of frequent contacts, while social loneliness reduced social networks [62]. In addition to the impact of the pandemic, our results support the relationship between loneliness and stress among expatriates, as in other studies conducted on the general population and students [9, 12–15].

We found that a lack of trust increased the loneliness of expatriates, which, in turn, raises their perceived stress levels. Additionally, trust in an individual is more likely to be associated with stress and loneliness compared to trust in institutions. Our findings support a previous study that discovered a negative correlation between stress, trust issues, and loneliness whereas loneliness mediated trust and stress [64]. Similar to this study, other research has found loneliness to be a moderating factor between stress and other variables [65, 66]. The evolutionary theory of loneliness suggests that it as an adaptive mechanism that produces an "aversive state" eliciting social pain, feeling of being unsafe, heighten threat sensitivity, and a negative social information bias [12, 16]. These factors deteriorate social and personal attachment [16, 17]. This model explains how loneliness integrates a person's sense of being unsafe and triggers an anarchy mechanism within their mind. Consequently, individuals become more sensitive to threats and attacks [13]. This later incorporates trust issues in the form of social connectedness, as it implies sensitivity to different cultural contexts (5, 22, 67–69). In many of these cases, use of social media and wide access of internet and communication technology can reduce these aversive states- including loneliness. Besides, respected organizational support during the world-wide crisis moment, transparent communication process, evaluation of the mental health condition along with their general health and provide local and international support intervention could be very helpful to them to overcome these mental conditions.

### Strengths and limitations

The study results have several strengths and a few limitations. One of the strengths is that we explore the social and cognitive psychology of expatriates, a relationship that has not been studied in literature or theory, especially during a crisis. However, the novelty of these findings underpins the social-cognitive psychology of expatriates in any situation, as psycho-social adjustment is a continuous process. Another strength of this study is the large sample size of the expatriates covering all countries worldwide. Additionally, the scale used for measuring loneliness, stress, and trust are widely acceptable, which added more strength-based findings. Furthermore, our study integrates the attachment component of adults with the cognitive and evolutionary perspective of loneliness to produce output. Nonetheless, the study has several limitations. Most of the respondents are female (> 70%) and students (> 70% had a college degree), which may lead biased results and a reflection of their social-cognitive psychology. Another limitation is that all results were estimated using secondary data collected online, which is not in the hands of the researchers, leading to an uneven sample of expatriates.

## Conclusion

The mental well-being of expatriate is dependent on recent circumstances and the psychological characteristics affected by these circumstances. The dynamics of attachment and the evolutionary cognitive theory of loneliness together describe their stress during the critical moments. Interventions that increase trust factors indirectly mitigate stress by addressing behavioural manifestation of concealed loneliness. Besides, creating a strong linkage among migrants as well as migrants with the local community are important to ensure proper mental wellbeing of expatriates. As this is the first study on this topic, further empirical research may be necessary to establish these findings for migrants and individuals who have lived in challenging cross-cultural situations. Perhaps, this study findings can be used by the relevant stakeholders and migrant communities and host countries to tackle the mental health conditions of expatriates (or migrants) by building up an interactive system/intervention with the hosted communities.

## Supplementary Information

**Additional file 1**. Supplementary file.

## Data Availability

The data was openly available in the open science forum (OSF)—available at https://doi.org/10.17605/OSF.IO/Z39US

## Declarations

### Geolocation information

Globally 179 countries.
