# Supplementary material for: Loneliness and trust issues reshape mental stress of expatriates during early COVID-19: a structural equation modelling approach
Source: BMC Psychol. 2023 Apr 29;11:140. doi: 10.1186/s40359-023-01180-9 (PMC10148631; doi:10.1186/s40359-023-01180-9)
Supplement: Supplementary file 1 — Additional file 1. Supplementary file. [file 40359_2023_1180_MOESM1_ESM.docx]

**Supplementary file**

**Perceived stress: PSS-10 scale** (ranked as 1-never, 2-almost never, 3-sometimes, 4-fairly often, 5-very often)

In the last week, how often have you-

1. been upset because of something that happened unexpectedly?
2. felt that you were unable to control the important things in your life?
3. felt nervous and “stressed”?
4. felt confident about your ability to handle your personal problems?
5. felt that things were going your way?
6. found that you could not cope with all the things that you had to do?
7. been able to control irritations in your life?
8. felt that you were on top of things?
9. been angered because of things that were outside of your control?
10. felt difficulties were piling up so high that you could not overcome them?

**Perceived loneliness: PLS-3 scale** (ranked as 1-never, 2-almost never, 3-sometimes, 4-fairly often, 5-very often)

In the last week, how often have you-

1. felt that you lacked companionship?
2. felt left out?
3. felt isolated from others?

**Trust issues** (rank on 0 as do not trust to 10 as have complete trust)

Interpersonal trust

1. In general, how much do you trust most people?
2. In general, how much do you trust most people you know personally

Institutional trust

How much you personally trust each of the institutions below-

1. Country’s parliament/government?
2. Country’s police?
3. Country’s civil service?
4. Country’s health system?
5. The World Health Organization (WHO)?
6. Country’s government’s effort to handle Coronavirus?
